# Brown Recluse Spider Bite Resulting in Coombs Negative Hemolytic Anemia in a Young Male Requiring Blood Transfusion

**DOI:** 10.7759/cureus.26574

**Published:** 2022-07-05

**Authors:** Usama Talib, Ahmed H Abdelfattah, Maryam Talib, Hadeel A Dawoud, Nidda Ayub, Sania Ayub, Amaar Talib

**Affiliations:** 1 Internal Medicine, University of Kentucky College of Medicine, Lexington, USA; 2 Hospital Medicine, University of Kentucky College of Medicine, Lexington, USA; 3 Internal Medicine, Basic Health Unit, Hajiwala, PAK; 4 Critical Care Medicine, Mansoura University, Mansoura, EGY; 5 Internal Medicine/Hematology, Sharif Medical and Dental College, Lahore, PAK; 6 Internal Medicine, Quetta Institute of Medical Sciences, Quetta, PAK; 7 Orthopaedics, Hijaz Hospital, Lahore, PAK

**Keywords:** symptomatic anemia, bleeding and blood products, internal med, clinical hematology, delayed hemolytic anemia, coomb's negative, hemolytic anemia, loxosceles bite, brown recluse spider bite, spider bite

## Abstract

Spider bites, including the bites of recluse spiders (*Loxosceles*, also known as brown spiders), usually lead to local symptoms; however, severe systemic symptoms have also been reported in the literature. Management of spider bites is based on symptoms. In severe cases involving the development of angioedema, hemolytic anemia, skin necrosis with superimposed bacterial infection or disseminated intravascular coagulation, antibiotics, steroids, blood transfusions, and plasma exchange may also play a role. We present a case of a brown recluse spider bite resulting in symptomatic hemolytic anemia and jaundice requiring blood transfusion.

## Introduction

North and South America, in particular, is known to be home to several spider species [1]. Diagnosis of a spider bite is commonly made by observing the spider during or immediately after the bite. One way to identify the brown recluse spider is by counting its eyes. Brown recluse spider has six compared to eight eyes of other spider species. Spider bites are usually painless but can lead to local and, in rare cases, systemic symptoms. 

In the case of a brown recluse spider bite, immediate local symptoms include pain and a burning sensation at the site of the bite [2]. Pain gradually worsens over the next several hours, and a blister forms at the bite site, which can take up to a week to heal [3]. Necrosis at the site is reported in 10%-20% of cases [4]. Systemic effects can range from fever, malaise, myalgias, and jaundice, while more severe cases can additionally result in rhabdomyolysis, hemolytic anemia, disseminated intravascular coagulopathy (DIC), angioedema, myonecrosis, and even death [2,5]. The incidence of severe symptoms resulting from a brown recluse spider bite is less than 1% [5].

Hemolytic anemia from a brown recluse spider bite can result in a positive direct Coombs test [6]. Elevated lactate dehydrogenase (LDH) level, total bilirubin and indirect bilirubin and low haptoglobin levels are helpful for diagnosis of hemolytic anemia. A combination of elevated LDH level and serum total bilirubin is a useful indicator of developing hemolytic anemia, with high sensitivity (94%) and specificity (91%) [7]. Fluids and analgesics are commonly used to treat spider bites resulting in pain, malaise and mild fever. Blood transfusion may be required for severe hemolytic anemia.

## Case presentation

We present a 19-year-old male with no previous medical history who presented to the emergency department (ED) with a two-day history of pain and swelling in his right forearm. The pain and swelling were accompanied by nausea, vomiting, and fevers. The symptoms started with pain following a brown recluse spider bite; the patient was able to identify the spider species immediately after feeling the bite. He presented to the ED the day before admission for milder symptoms and was discharged after supportive care with fluids and analgesics. Despite taking two doses of prescribed Trimethoprim/Sulfamethoxazole (prescribed due to concern for superimposed bacterial skin infection at the site of bite), the patient returned due to worsening erythema, pain, nausea, and subjective fevers. 

**Figure 1:**
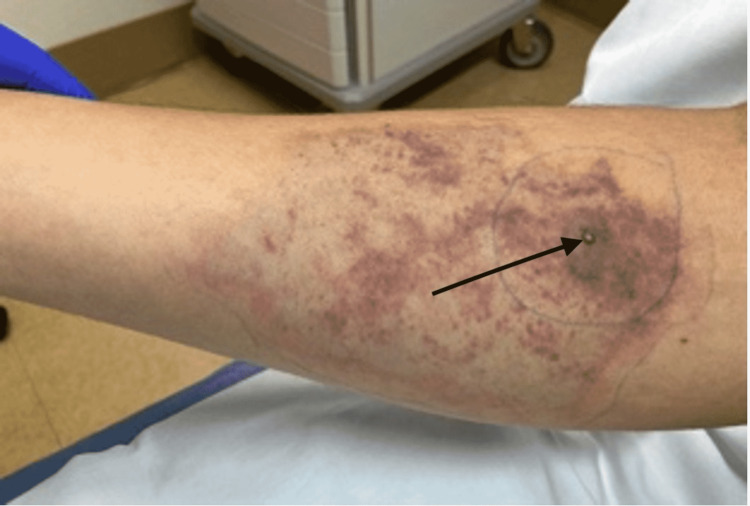
Vesicle at the site of brown recluse spider bite (black arrow) surrounded by ecchymosis and erythema.

The patient was hemodynamically stable upon presentation to the ED and received IV fluids for dehydration due to inadequate oral intake due to nausea and reported fevers. Laboratory tests (labs) were notable for an international normalized ratio (INR) of 1.5, a serum total bilirubin of 2.1, conjugated bilirubin of 0.5, and a creatinine kinase (CK) of 477, and creatinine (Cr) of 1.34 (from 0.9). Hemoglobin (Hb)/Hematocrit (Hct) was 16.4/46.9. Examination revealed dry mucous membranes, a central vesicle at the site of the bite surrounded by a rim of developing skin necrosis and erythema (Figure 1). The patient was admitted to medicine service, and a toxicologist was consulted for further recommendations. The patient was started on IV antibiotics and oral prednisone daily, as recommended by the toxicologist. His labs were monitored during his stay for worsening signs of hemolysis and rhabdomyolysis or the development of disseminated intravascular coagulation (DIC). His labs, including creatine (Cr) and creatine kinase (CK), had normalized by the end of his first day in the hospital. Before discharge, the ecchymoses and erythema surrounding the bite wound had started to reduce in size. The patient was discharged on corticosteroid taper and oral antibiotic for cellulitis.

Five days later, the patient presented to the ED again with complaints of yellow discoloration of the eyes. He also reported dizziness, fatigue, and dyspnea. He denied palpitations, weakness, orthostasis, and chest pain. His right forearm wound had improved. On examination, he was jaundiced with scleral icterus and yellowing of the forehead and face. His labs were consistent with acute hemolytic normocytic anemia. His Hb/Hct dropped from 16.4/46.9 (last admission) to 9.7/28. LDH was found to be high at 830, bilirubin was at 6.3 (mainly indirect), and aspartate aminotransferase (AST)/alanine transaminase (ALT) was mildly elevated at 72/58. A urine drug screen was negative, but urinalysis showed a large amount of blood with no red blood cells (RBCs) on microscopic analysis of urine, signifying hemoglobinuria. Infectious workups, including blood culture, urinalysis, chest X-ray, influenza, COVID-19, HIV, and hepatitis screen, were negative. Reticulocyte count increased from 0.6 (last admission) to 9.1, haptoglobin was low < 10 (compared to normal on the last admission), with a normal platelet count and international normalized ratio (INR). A test for glucose 6-phosphate dehydrogenase (G6PD) deficiency was unremarkable, and a Coombs test was negative. The hematology team was consulted, and his hemolytic anemia was attributed to the brown recluse spider bite. The patient was managed conservatively on the medical floor with hydration and one-unit packed red blood cells (PRBC) transfusion due to his low Hb of 6.7. Subsequently, his Hb, LDH, bilirubin and ALT/AST started to normalize. The patient was discharged home after five days with instructions to follow up with his primary care physician (PCP).

## Discussion

Bites from insects, including spiders, are a rare cause of hemolytic anemia. Most symptoms, if any, from spider bites are mild and resolve without intervention [4]. Identification of the spider at the time of the bite or immediately afterward is the most common diagnostic method. Coombs-positive hemolytic anemia in patients due to insect bites, including spider bites, has previously been reported [8]. In this paper, we report a Coombs negative hemolytic anemia and rhabdomyolysis in a patient due to a brown recluse spider bite. Clinically significant hemolytic anemia and bilirubinemia from a spider bite usually occur early in the course of the disease; however, in our patient, the anemia and bilirubinemia occurred seven days after the bite [9]. Due to possible systemic toxicity from a brown recluse spider bite, workup for DIC should be considered in such cases.

Even though most symptoms are mild and self-resolving, symptomatic treatment is recommended for moderate to severe cases [4]. Antivenom, although not commonly available, has been reported to be beneficial in the treatment of South American recluse spider bites [9]. Hemolytic anemia, though rare, can be severe enough to require a blood transfusion. Spider bites can also rarely lead to the development of DIC, which may require plasma exchange and, therefore, needs to be kept in mind during management. In the pediatric population, symptoms may be more severe, including hemolytic anemia, renal failure, and hypotension [10]. In one reported case of a patient with severe hemolytic anemia and a Hb level of less than two, plasma exchange was beneficial [11]. Rhabdomyolysis associated with spider bites is usually treated with hydration and ensuring adequate urine output. 

Vascular ulcers, diabetic foot ulcers, and skin infections, including cellulitis, may appear similar to spider bites and need to be kept in the differentials. The enzymes and toxins contained in the spider venom are responsible for the local and systemic signs of spider bites [12]. Spider bites can lead to systemic signs, including, but not limited to, severe hemolytic anemia, rhabdomyolysis, and hypotension, which can lead to organ failure and even death. In areas with a high prevalence of spiders and a high incidence of spider bites, familiarity with systemic toxicity following a spider bite and possible treatment options is prudent.

## Conclusions

In rare cases, spider bites can lead to systemic symptoms, including hemolytic anemia, that may or may not be Coombs positive. Insect bites, including spider bites, must be kept in the differential of unclear causes of hemolytic anemia. Management of spider bites is based on symptoms with intravenous fluids, blood transfusion (if needed), and, in some cases, steroids. Plasma exchange is an option in severe cases refractory to symptomatic management.
